# Familiarity Detection and Memory Consolidation in Cortical Assemblies

**DOI:** 10.1523/ENEURO.0006-19.2020

**Published:** 2020-05-08

**Authors:** Xiaoyu Zhang, Fang-Chin Yeh, Han Ju, Yuheng Jiang, Gabriel Foo Wei Quan, Antonius M.J. VanDongen

**Affiliations:** 1 Program for Neuroscience and Behavioral Disorders, Duke-NUS Medical School, 169857, Singapore; 2 NUS Graduate School for Integrative Sciences and Engineering, National University of Singapore, 117456, Singapore

**Keywords:** consolidation, microcircuits, neuronal network, optogenetics, recognition memory

## Abstract

Humans have a large capacity of recognition memory (Dudai, 1997), a fundamental property of higher-order brain functions such as abstraction and generalization (Vogt and Magnussen, 2007). Familiarity is the first step towards recognition memory. We have previously demonstrated using unsupervised neural network simulations that familiarity detection of complex patterns emerges in generic cortical microcircuits with bidirectional synaptic plasticity. It is therefore meaningful to conduct similar experiments on biological neuronal networks to validate these results. Studies of learning and memory in dissociated rodent neuronal cultures remain inconclusive to date. Synchronized network bursts (SNBs) that occur spontaneously and periodically have been speculated to be an intervening factor. By optogenetically stimulating cultured cortical networks with random dot movies (RDMs), we were able to reduce the occurrence of SNBs, after which an ability for familiarity detection emerged: previously seen patterns elicited higher firing rates than novel ones. Differences in firing rate were distributed over the entire network, suggesting that familiarity detection is a system level property. We also studied the change in SNB patterns following familiarity encoding. Support vector machine (SVM) classification results indicate that SNBs may be facilitating memory consolidation of the learned pattern. In addition, using a novel network connectivity probing method, we were able to trace the change in synaptic efficacy induced by familiarity encoding, providing insights on the long-term impact of having SNBs in the cultures.

## Significance Statement

Studies of memory mechanisms in neuronal networks formed by cultured neurons have been complicated by spontaneously occurring synchronized network bursts (SNBs), which prevent encoding of stimulus information. We have developed an optogenetic method to suppress SNBs using random dot stimuli, which allowed us to demonstrate the ability of cultured cortical networks to detect a familiar input. Whereas SNBs interfere with familiarity encoding, they may facilitate memory consolidation. These results indicate that generic cortical microcircuits have an innate ability for familiarity detection, a form of recognition memory.

## Introduction

Learning and memory are indispensable for brain functionality. Recognition, the ability to recognize previously experienced sensory inputs, is a form of declarative memory that is common in our daily life and fundamental to many higher-order processes such as concept learning, abstraction and generalization. Prior studies (Nickerson, 1965; Shepard, 1967; Standing, 1973) have demonstrated that subjects can recognize many thousands of pictures several days after they have seen the pictures only once, suggesting a vast recognition memory for images, with no capacity limit detected in practical experiments.

Familiarity and recollection are two components of recognition memory (Yonelinas, 2002; Squire et al., 2007). Recollection requires accurate recall of details with their context, whereas familiarity merely needs a signal indicating that the object has been encountered before (Wixted, 2007). Unlike conditional or reinforcement learning, which requires association or reward, familiarity is simply encoded through unsupervised learning (i.e., sensory experience) and retrieved on re-presentation of the input. These features have made familiarity a perfect paradigm to study learning and memory from a bottom-up perspective.

Synaptic plasticity has long been implicated in the processes of learning and memory. Memories are thought to be encoded and stored in “engrams,” groups of neurons connected through synapses whose efficacy was modified during learning. Characterization of the changes in a population of neurons following learning and after memory formation can be an important building block to forge a link between molecular/cellular synaptic plasticity and behavioral modifications.

Dissociated neuronal cultures growing on multielectrode arrays (MEAs) provide an accessible way to study familiarity in neuronal populations *in vitro*. MEAs are designed to obtain parallel recordings at multiple sites of the dynamics of cultured neuronal networks. By introducing the light-gated ion channel channelrhodopsin-2 (ChR2) into neurons, non-invasive optogenetic stimulation has been made possible for encoding sensory experiences. Correlation between sensory input and network activity modification can be established to provide insights into the mechanisms of learning and memory at the neuronal network level.

A few groups have already made efforts to study learning and memory using MEAs. Maeda et al. (1998) and Jimbo et al. (1998) found that the reliability and reproducibility of stimuli-evoked network bursts was enhanced after tetanization at single electrodes. Tateno and Jimbo (1999) reported improved temporal precision of initial response spikes to test stimuli after training. Later Jimbo et al. (1999) reported that single-site stimulation could induce pathway-dependent potentiation or depression. However, subsequent attempts made to reproduce these observations were less successful. By including control recordings, Wagenaar et al. (2006b) argued that the “positive modifications” observed did not differ significantly from what could be caused by the drift in spontaneous network activity. Applying the same tetanization protocol (20 Hz delivered at a single site, as in Jimbo et al., 1999), Chiappalone et al. (2008) failed to observe significant potentiation; instead, they saw only a global decrease in the evoked network activity (Chiappalone et al., 2008, their Fig. 4). Additional studies were conducted, either with electrical (Massobrio et al., 2015) or optogenetic stimulation (Lignani et al., 2013). Constrained by the inflexibility of input and output (electrical stimulation and data collection through the same electrodes), most of the studies characterized network dynamics by measuring changes in mean firing rate, as well as burst frequency and duration, the values of which are highly variable and poorly associated with learning. Consequently, results from these studies have not reached a common agreement to date. Whether *in vitro* neuronal networks exhibit learning and memory as an emerging property remains inconclusive.

Computer simulations with neural network models previously demonstrated that generic cortical microcircuits with bidirectional synaptic plasticity can perform familiarity detection (Zhang et al., 2017). However, these simulated networks do not have all the properties of neuronal networks growing on MEAs. One major difference is the synchronized network bursts (SNBs), which are universally observed in biological cultures. A SNB is characterized by network-wide synchronized high-frequency firing that is spontaneously initiated and lasts several hundred milliseconds. SNBs occur periodically in cultured cortical networks in the absence of external inputs, and are separated by windows of nearly silent network activity with occasional sparse asynchronous firings (Maeda et al., 1995; van Pelt et al., 2004; Eytan and Marom, 2006; Chiappalone et al., 2007; Colombi et al., 2016). *In vitro* studies investigating the capability of neuronal networks to process spatiotemporal inputs have shown that SNBs disrupt short-term memory (Dranias et al., 2015; Ju et al., 2015). Similarly, when SNBs were introduced in simulated neural networks, average performance on familiarity detection declined (Zhang et al., 2017). In this study, we provide evidence on familiarity detection in MEAs in the presence of SNBs.

## Materials and Methods

### Culture preparation

Dissociated cortical primary cultures were prepared from Sprague Dawley rat embryonic day (E)18 brains from either sex. Cortical tissue was dissected in ice-cold HBSS. The isolated cortices were digested using the Worthington Papain Dissociation System (Worthington Biochemical Corporation). Cells were plated on MEA dishes with 252 electrodes arranged in a 16 × 16 grid (30-µm diameter, 200-µm interelectrode distance, Multi-Channel Systems). Prior to plating, MEAs were cleaned with 1% Tergazyme solution, sterilized with 70% ethanol, surface-treated with fetal bovine serum (FBS), and coated with 0.1 mg/ml poly-D-lysine (Invitrogen). Cells were plated onto a circular area with a diameter of 6 mm centered at the electrode array area. The final density was ∼4000 cells/mm^2^. The plating droplet was left on the MEAs for 30 min to allow cell attachment. Culturing medium (Neurobasal supplemented with 2% B-27, 0.5 mM L-glutamine, 10% penicillin/streptomycin) with 10% FBS (Sigma) was then added to the cells (1 ml for each MEA). Medium was completely changed 24 h later [1 d *in vitro* (DIV1)] to remove FBS. Subsequent medium changes were done on DIV6, DIV10, and every 3 d afterwards. Half of the medium was replaced each time. Cultures were covered with plastic caps with a fluorinated ethylene-propylene membrane (ALA-Scientific) and maintained in a humidified CO_2_ incubator (5% CO_2_, 37°C). On DIV9, MEA cultures were transfected with an adeno-associated virus (AAV9) encoding ChR2 (AAV9.hSyn.hChR2(H134R)-EYFP.WPRE.hGH, Addgene 26973P, MOI 100,000). Half of the medium was changed on DIV10 to prevent virus toxicity. Alternative transfection on DIV1 was applied on cultures used in the study of early connectivity development. Overall, we observed similar development patterns between cultures transfected on DIV1 and DIV9. DIV9 was chosen as the transfection date for cultures used for the main experiments for its least disruption on long-term viability of the cultures. All experimental procedures were conducted in accordance with Institutional Animal Care and Use Committee (IACUC) and approved by SingHealth.

### MEA recording

Recordings were performed on an anti-vibration table and in a Faraday cage. During recordings, MEAs with culturing medium (see above, Culture preparation) were placed in a customized CO_2_ incubator placed on top of an inverted microscope (see below, Optical stimulation) inside the cage. Extracellular electrophysiological signals were acquired using the USB-MEA256 hardware systems (Multi Channel Systems). MC_Rack software (Multichannel Systems) was used to process extracellular signals that were high pass filtered at 300 Hz and low pass filtered at 3 kHz with fourth-order Bessel filters. Each channel was sampled at a frequency of 20 kHz. Action potentials were detected using a voltage threshold set at six times the SD (6σ) of the biological noise for each recording channel.

### Optical stimulation

Stimulus presentations and MEA recordings were triggered and synchronized by transistor-transistor logic (TTL) pulses generated by MATLAB that signaled the beginning and end of each session. Optical stimulation was conducted using a 500-mW DPSS laser with a wavelength of 473 nm. The laser beam was optically expanded and projected onto a reflective spatial light modulator (SLM; Holoeye Photonics), with a resolution of 1920 × 10^80^ pixels. The patterns reflected by the SLM were controlled by the DVI graphics output of a personal computer and were refreshed at 50 Hz. The reflected light patterns were then projected onto MEA cultures through the objective lens of an inverted microscope (Eclipse Ti-E, Nikon). The final light intensity at the MEA culture is ∼4.5 mW/mm^2^. This setup allowed us to design arbitrary blue-light images and use them as stimuli. The stimulation presentation was programmed with the Psychtoolbox-3 (http://psychtoolbox.org) in MATLAB.

### Cross-correlation histogram (CCH) probing

Cultured neuronal networks were probed with optical stimuli to identify synaptic connections and monitor changes in their efficacies. The optical stimulation area, which approximately covered the electrode array area, is divided into a 16 × 16 grid, resulting in 256 square “dots.” Each probing session is conducted by stimulating the culture with a series of random-dot frames, in which all grid positions were black, except for five randomly-selected positions which were white, resulting in five blue dots simultaneously stimulating the network when the laser is on. Each stimulating frame lasts 100 ms, during which the light is on for 40 ms, and off for 60 ms. Each of the 256 positions in the grid is stimulated 20 times in total in a random order during a probing session, resulting in a random dot movie (RDM) of 5120 frames. Each probing session lasts ∼1.7 min. Neuronal responses were recorded from the 252 MEA electrodes concurrently with the optogenetic random dot probing. A potential causal relationship between each stimulating grid position and each electrode (recording position) was evaluated by calculating a CCH between the electrode response time-series and the stimulating time-series. CCHs are calculated as

$$cch\left(\tau \right)=\underset{t}{\sum }i\left(t\right)j(t-\tau ),$$

where τ is the time lag between the two time-series, and i, jare the time-series representations (1-dimensional vectors) of the electrode response time points and the stimulus time points. The entire probing session was binned into 1-ms windows. For an electrode, if it detects spikes in a particular time bin, the time-series vector i will contain 1 in the corresponding time bin, otherwise 0. For a stimulating position, if a probing stimulus is imposed in a particular time bin, the time-series vector j will contain 1 in the corresponding time bin, otherwise 0. CCH calculates the discrete cross-correlation between the two time-series and find the time lag that returns the largest correlation (the peak of the CCH curve). A sharp peak with a positive time lag in the CCH indicates that the electrode consistently detects a response at a defined time window after the grid position is stimulated, and therefore a directional synaptic connection likely exists between the grid position (presynaptic) and the electrode (postsynaptic). Assuming the recorded spike train (electrode response) follows a Poisson process, the significance of the peak can be tested against the *p* value of the Poisson distribution:

$$P\left(k\right)=e^{-\lambda }\frac{\lambda^{k}}{k!}.$$

P(k)computes the chance of observing a peak value of k given the mean of cch(τ) as λ. When the chance falls below P = 1e^−6^, and the electrode detects a response in more than half of the trials (10 out of 20 times when the dot is stimulated), we draw a connection. The value 1e^−6^ is chosen empirically to control the number of connections that will be detected as reliable connections in a culture. Setting it too high (1e^−5^) will result in having too many connections detected, e.g., some channels are constantly firing and thus will be assigned connections with nearly all stimulating position. Setting it too low (1e^−7^) will result in having too few connections detected and increasing the variance of network analysis. The latency of the connection is reflected by the time lag $\tau_{p}$ where the CCH reaches its peak after stimulation. The efficacy of the connection is estimated by the area under the poststimulus time histogram (PSTH). With the detected connections, we were able to draw a connectivity map for each culture. The connectivity map comes together with directionality information, which can be used to augment analysis and understanding. Two connectivity maps of the same culture obtained before and after a certain treatment can be compared with evaluate whether the culture has undergone significant connectivity changes. For instance, the relative change of the efficacy, change in the area under the PSTH curves divided by area under the initial PSTH curve, is calculated for all connections. Positive efficacy change indicates potentiation, while negative efficacy change indicates depression. To evaluate the overall network efficacy change, the positive changes and negative changes are summed together respectively over all connections and represented as “P: ∑(0 < changes < 100% + changes > 100%)” and “N: ∑(changes < 0%)” in the figure legend. The unit of P and N is [%]. Positive changes > 100% are capped at 101%. The potentiation to depression ratio “R” (R = P/|N|, where |N| is the absolute value of N) is calculated to reflect the overall change in network efficacy. R > 1 indicates long-term potentiation (LTP) at the network level and R < 1 indicates long-term depression (LTD) at the network level. CCH probings were conducted 5 min after each treatment to reflect the long-term effect associated with the treatments.

### Experimental design

**Figure 1.**
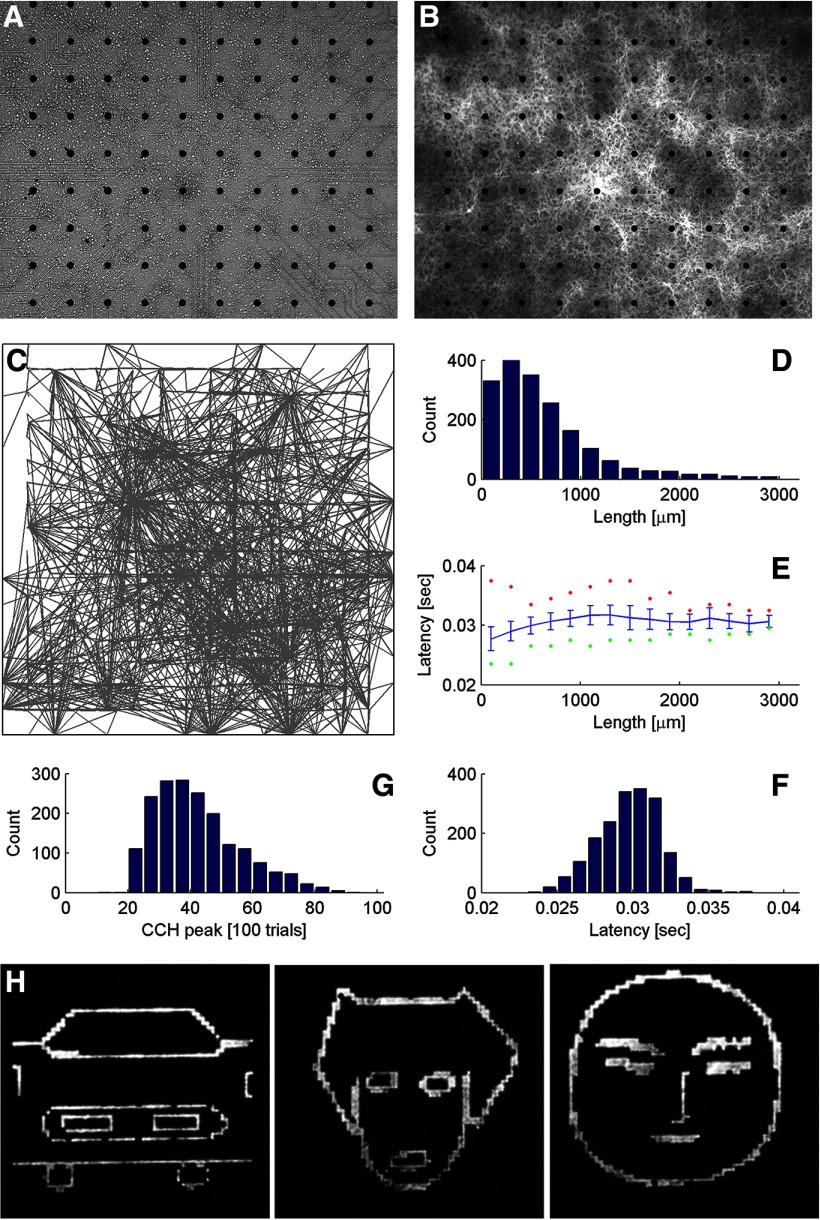
Network connectivity and stimulus pattern. ***A***, ***B***, Pictures of a DIV30 MEA culture taken with a 4× objective illuminated by bright-field light (***A***) and fluorescent light (510 nm with 3-s exposure time) to visualize ChR2-YFP expression (***B***). ***C–G***, Network connectivity summary of the MEA culture. ***C***, All significant connections detected by CCH probing. Each line is drawn from a presynaptic dot position to a postsynaptic electrode. ***D***, Histogram of the distances of the connections in ***C***, binned at 200 µm, which is the spacing between two adjacent electrodes. ***E***, Group average latency of the corresponding connections in ***D***. Error bars indicate the SDs. Max and min latencies are marked as red and green. ***F***, Histogram of the latencies of the connections. ***G***, Histogram of the CCH peak values of the connections. The value is normalized to 100 trials. A peak at 100 means for every trial when the presynaptic dot position was stimulated, the postsynaptic electrode detected a response. Connection efficacy is positively correlated with the CCH peak, but to be more precise, we used the area under the PSTH curve as the measure of connection efficacy in the subsequent analyses. ***H***, Cartoon-like images of a car front, a dog face and a human face, projected as blue light patterns onto an MEA culture with ChR2-YFP expressing neurons.

We used three cartoon-like images of a car front, a dog face and a human face (Fig. 1*H*) to study the networks’ ability to learn and memorize complex input patterns. The three patterns were converted to line drawings in 50 × 50 grids of the same size as the stimulating area. The luminance of each pattern is tuned to balance the elicited neuronal baseline response. Experiments typically consisted of three sessions: baseline recording, learning stimulation and testing recording. Network baseline responses to each of the three patterns was recorded 10 times. Three patterns were presented in random orders with 10-s intervals and 100-ms stimulation time. As the network response is subject to noise introduced by fluctuation in the network background activity level due to SNBs, applying an illuminating window of 100 ms enables us to record stable and consistent network responses for baseline and testing phases. Learning was conducted by stimulating the cultures with one of the three patterns at 50 Hz (50% duty cycle, 10 ms on and 10 ms off) for 60 trials with 9-s intervals and 1-s stimulation time. Network testing (postlearning) response was recorded the same way as baseline recording.

### Burstiness index (BI)

We adopted the idea of BI (Chiappalone et al., 2005; Hinard et al., 2012) to quantify degree of network bursting. Continuous recordings of network spontaneous activity (10 min, recorded at the beginning of experiments) were divided into 1-s bins and the fraction of spikes contained by the 15% most active bins was calculated (f15). BI was defined as BI = (f15 – 0.15)/0.85, with 0 for not bursting at all, and 1 for maximum burstiness. The 15% was chosen empirically with the observation that SNBs occur on average in 15% of the time bins in an SNB-dominant culture. Equivalently it assumes the culture has an average interburst interval (IBI) of 6.7 s. For cultures with average IBI <6.7 s, we increased the 15% accordingly.

### RDMs

In order to suppress SNBs, neuronal networks were pre-stimulated by RDMs. In experiments involving RDM treatment, the movie was generated in a 10 × 10 grid of the same size as the stimulating area. In each frame, 25 out of 100 dots were randomly chosen and illuminated to stimulate the culture for 50 ms. The culture was then allowed to rest for 50 ms in dark. Frames were refreshed at a frequency of 10 Hz. These values for the frequency and number of dots were determined to be optimal for suppression of SNBs. RDMs were played for 0.5–1 h to the cultures, after baseline recording, and before learning stimulation.

### Culture exclusion

For experiments comparing the conventional learning paradigm and the RDM-learning paradigm (Figs. 7*G*,*H*, 8), we started with 26 healthily plated cultures (enough cells survived by DIV22 for good coverage of the stimulating area). Before each experiment, we recorded a 10-min session of the culture’s activity at rest and excluded cultures with frequency of background SNBs >0.2 Hz (too active) or <0.05 Hz (too quiet). Five cultures were excluded this way. Cultures whose network efficacy changed significantly before and after the baseline/testing recording phase were also excluded, because they were found to be unstable and produce inconsistent results in other steps. Five cultures were excluded this way. For the RDM learning paradigm, cultures whose bursting activity was not significantly suppressed by the RDMs (R > 0.2) were excluded from the study, because their subsequent learning capacity were compromised. Three cultures were excluded this way. Eventually we were left with a total of thirteen cultures: the conventional learning paradigm was used for five cultures, in three cultures we used the RDM-learning paradigm and conducted testing 10 min after the learning stimulation, while in five cultures we used the RDM-learning paradigm with extended testing at 30 min, 1 h, and 24 h after learning.

### Support vector machine (SVM) classification

Pattern-induced network responses at baseline and testing phases are binned at 10 ms. Data from all bins of the three classes (human, dog, car) are pooled together (pooling-all-sample method) to train one SVM classifier, to avoid the curse of dimensionality (the number of features is greater than the number of instances). The trained SVM classifier was then applied to classify 5-min continuous recordings of network spontaneous activity (binned at 10 ms) obtained before the baseline phase and immediately after the learning phase. Classified labels during SNBs were summarized to get the ratio of classified SNB bins belonging to each of the three complex patterns. In a separate effort to validate the usage of the pooling-all-sample method, 60% of the pattern-induced network responses were used to train the SVM classifier and the remaining data were classified by the trained classifier. LIBSVM (Chang and Lin, 2011, software available at http://www.csie.ntu.edu.tw/∼cjlin/libsvm, RRID:SCR_010243) and its built-in multiclass classification were used.

### Evaluation metric

The learning protocol (50 Hz, 50% duty cycle, 10 ms on, and 10 ms off for 60 trials with 9-s intervals and 1-s stimulation time) was applied equally to all cultures, because we did not set up any feedback loop that can be used to control the learning duration or stop the tetanus stimulation after the induced LTP exceeds a pre-set threshold. As a result, the learning-induced LTP might appear inadequate for some cultures, i.e., they may display similar magnitudes of change induced by “learning” as compared with response fluctuation due to noise. In light of this, the focus of the performance analysis is not the significance level of magnitude of change, but whether the learned pattern triggers more response than the control patterns at testing. In detail, the induced response differences between the familiar pattern and the two control patterns were averaged and then the value was normalized against the response to the familiar pattern to make the result comparable among cultures:

$$\Delta =\frac{\left(x_{f}-x_{c1})+(x_{f}-x_{c2}\right)}{2x_{f}},$$

where *x* is the network response to be evaluated. It can take the value of network firing rate induced by the input patterns or the ratio of classified SNB bins belonging to the input patterns [for more information, see above, Support vector machine (SVM) classification]. *x_f_* is the response induced by the familiar pattern. *x_c1_*, *x_c2_*are the responses induced by the two control patterns.

## Results

The goal of the experiments described here was to investigate the ability of cultured neuronal networks resembling cortical microcircuits to recognize a familiar (previously seen) stimulus pattern. We have used cultured cortical neurons growing on a MEA and transfected with ChR2, allowing us to optogenetically stimulating the network with complex patterns, while recording from hundreds of neurons (Fig. 1). A universal property of dense cultured cortical networks is the appearance of highly SNBs every few seconds (Maeda et al., 1995; van Pelt et al., 2004; Eytan and Marom, 2006; Chiappalone et al., 2007; Colombi et al., 2016). We have therefore characterized the development of SNB activity in our MEA cultures and developed an optogenetic approach to suppress them. In additional, we developed an optogenetic approach to define directional connectivity maps for the networks, using a CCH analysis, which allows us to measure alterations in synaptic efficacy following learning paradigms.

### Bursting, connectivity, and network development

**Figure 2.**
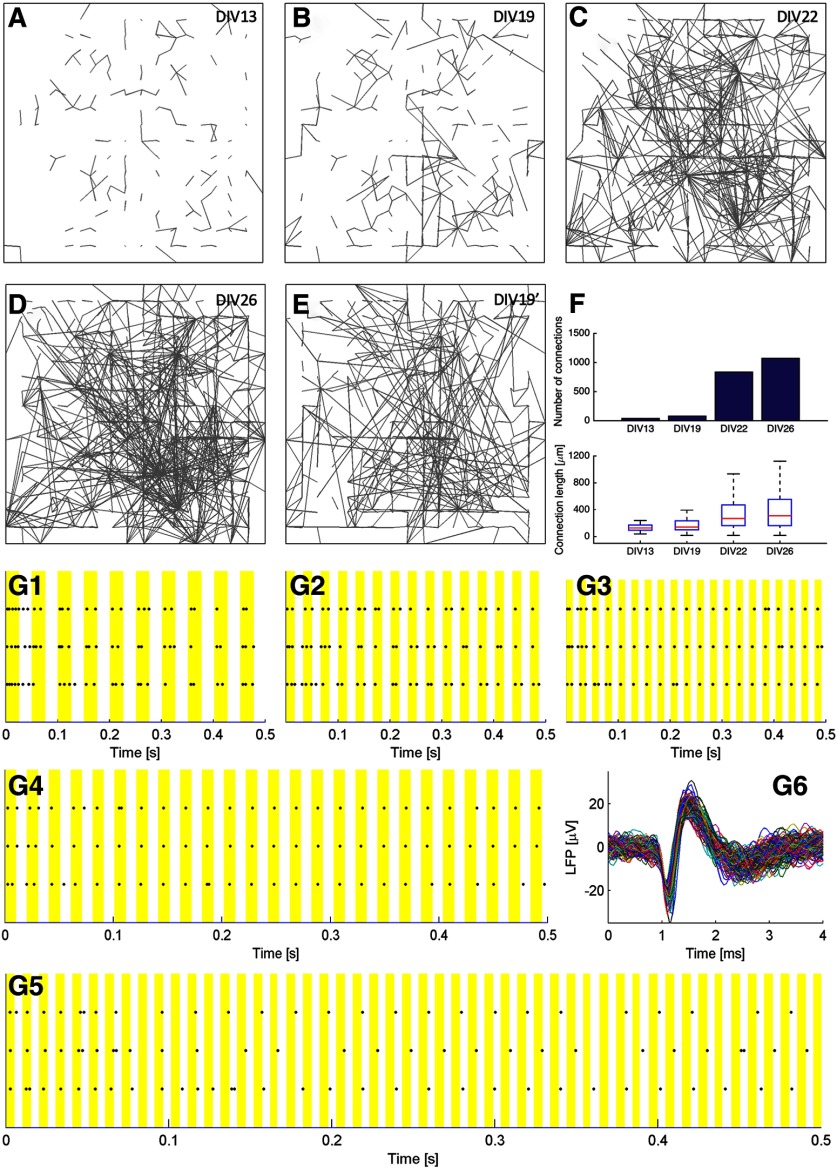
Development of network connections and neuronal responses to high-frequency stimuli. ***A–D***, Culture connectivity detected by CCH probing. The culture was transfected with ChR2-YFP on DIV1. CCH measurements were conducted on DIV13 (***A***), DIV19 (***B***), DIV22 (***C***), and DIV26 (***D***). ***E***, Culture connectivity if loosening the threshold of CCH detection on DIV19. ***F***, Summarizing the number of connections detected on DIV13, DIV19, DIV22, and DIV26 (**top**), and the distribution of connection length for each DIV (**bottom**). ***G1–G6***, Channel responses to light pulses of 20 Hz (***G1***), 30 Hz (***G2***), 40 Hz (***G3***), 50 Hz (***G4***), and 100 Hz (***G5***). ***G6***, Channel waveforms in response to 50-Hz pulses (***G4***). The channel is selected as a representative of channels with the best response to high-frequency tetanus. Culture was stimulated on DIV22. Yellow bars highlight when light pulses were delivered.

Similar to previous observations, SNBs emerged in the MEA cultures on DIV8 and became dominant from DIV13 onwards. The frequency and duration of SNBs increased over time and stabilized after DIV20 (Wagenaar et al., 2006a), with characteristic IBIs of 5–10 s. To trace the neuronal network development, we established longitudinal connectivity maps for the cultures (Fig. 2*A–D*). These maps reveal that local connections (length ≤ 300 µm) are detectable at DIV13 (Fig. 2*A*); a sparsely connected network starts to form by DIV19 (Fig. 2*B*); global connections (length > 300 µm) become widespread by DIV22 (Fig. 2*C*); finally, a densely connected network with extensive global and local connections is formed by DIV26 (Fig. 2*D*). If loosening the threshold used for CCH detection from 1e^−6^ to 1e^−5^ (see Materials and Methods for more details), long-range connections will appear in the connectivity maps at DIV13 (data not shown) and DIV19 (Fig. 2*E*).

The developmental profile described by Figure 2*A–D* varied 1 or 2 d for individual cultures with different cell densities. We performed most of our experiments from DIV22 onwards, so we could characterize learning-induced changes of a large number of connections and understand the effect of a training paradigm from the network perspective. This time window was also consistently used in (Chiappalone et al., 2008). A statistical summary of the number of connections and connection length of Figure 2*A–D* is provided in Figure 2*F*.

### Network response to high-frequency stimuli

Another benefit of using mature and strongly connected cultures is that they are able to fire more synchronously in response to high-frequency stimuli. The ChR2 version we used has the H134R mutation, which increases the photocurrent but is associated with slower channel-closure kinetics, resulting in a reduced temporal precision (Yizhar et al., 2011). It is shown in the CCH probing data that the latency of an optically elicited response is centered around 30 ms (Fig. 1*F*), which limits the firing frequency to be elicited through direct optical response to 30 Hz. Nevertheless, a subset of neurons in mature cultures was able to fire synchronously to stimuli with a frequency of >30 Hz (for examples, see Fig. *2G3–G4*). These neurons are usually receiving EPSPs from multiple concurrently stimulated grid positions. We name them as the “postsynaptic hub neurons” in connectivity maps. Strong connection efficacies in mature cultures assist the propagation of EPSPs, thereby securing the temporal precision of firing. Nevertheless, pyramidal neurons have been reported not to follow well with stimuli beyond 40 Hz, because they are limited by their intrinsic cellular biophysical properties (Hjelmstad et al., 1997; Gunaydin et al., 2010). The reliability of neuronal activation through ChR2 dropped significantly as the frequency of light pulse delivery increased (Arenkiel et al., 2007). We tested MEA culture responses to stimuli of 20–100 Hz (Fig. 2*G1–G5*). The highest frequency that cortical neurons could fire synchronously was 50 Hz. Therefore, we used 50 Hz as the learning stimulation protocol for subsequent experiments.

### Plasticity induction in presence of SNBs

**Figure 3.**
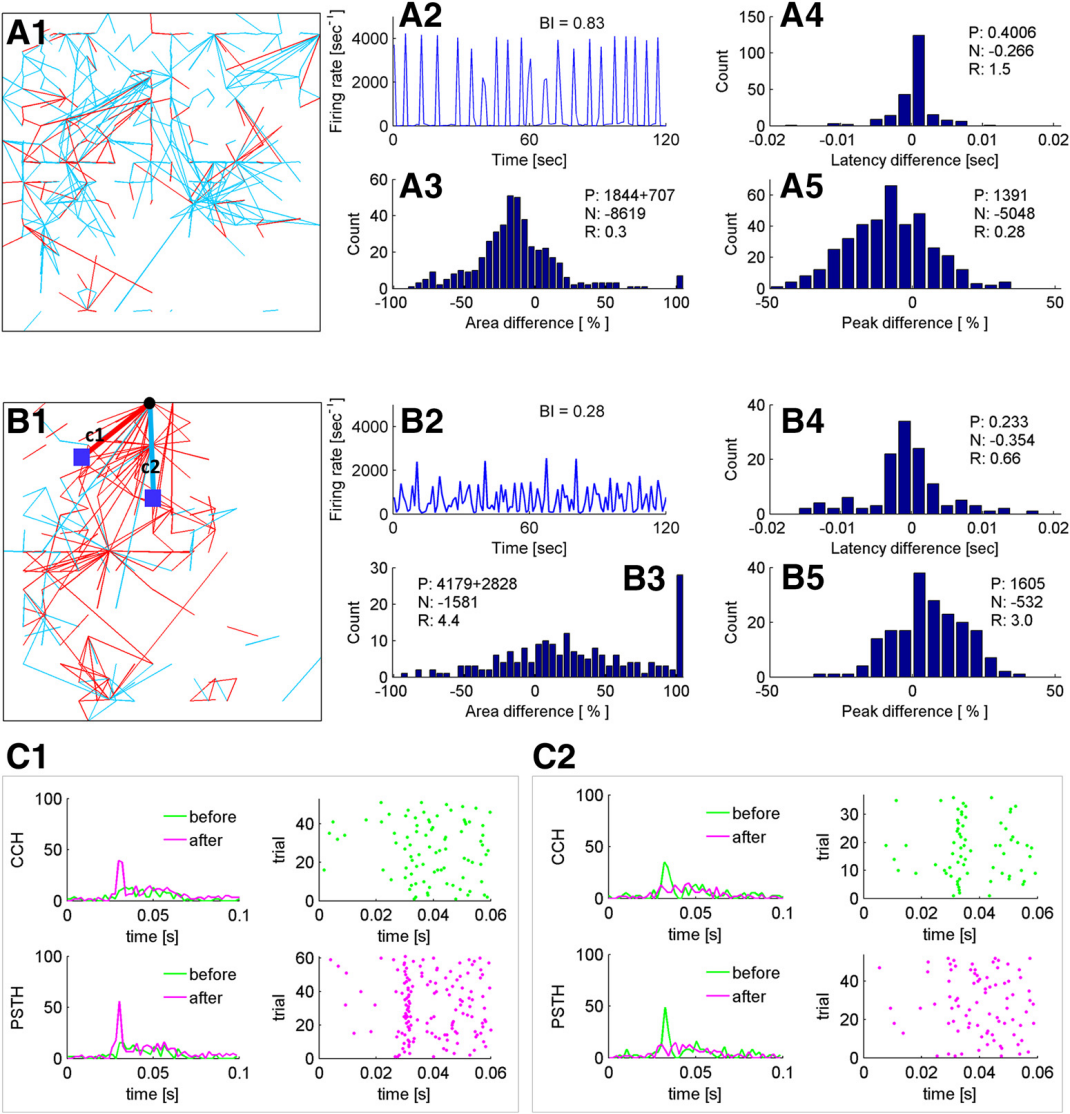
High-frequency tetanization induces LTD or LTP depending on culture “burstiness.” ***A1–A5***, Network connectivity change in a network with frequent SNBs. ***A1***, Potentiated (red) and de-potentiated (blue) connections in the culture detected by CCH probings conducted right before and 5 min after 50-Hz stimulation (whole field stimulation, 50 Hz for 60 trials with 9-s intervals and 1-s stimulation time). ***A2***, Network firing rate as a function of time, binning at 1 s. BI (see Materials and Methods) is 0.83, based on a 5-min recording of network spontaneous activity, conducted before any stimulation. ***A3***, Summary of efficacy change for all connections detected (see Materials and Methods, CCH probing, for more details regarding the calculation). P: ∑(0 < changes ≤ 100% + changes > 100%). N: ∑(changes < 0%). The unit of P and N is [%]. R reflects the overall change in network efficacy (R = P/|N|). ***A4***, Summary of the change in response latency for all connections detected. ***A5***, Summary of the change in PSTH peak heights for all connections detected. ***B1–B5***, Network connectivity change in a network with sparse SNBs (same format as ***A1–A5***). ***C1***, ***C2***, CCH, PSTH curves, and raster plots of the highlighted connections in ***B1*** before (green) and after (magenta) the 50-Hz stimulation. ***C1*** corresponds to connection c1, which was potentiated. The relative change of area under PSTH curve is 46.71%. ***C2*** corresponds to connection c2, which was depotentiated. The relative change of area under the PSTH curve is −20.06%. CCH curves were used to identify a connection and its response latency. PSTH curves were used to calculate the efficacy change.

By looking at the BI values, the cultures can be classified into two types. Cultures dominated by SNBs are found to be associated with high BI values [BI = 0.86 ± 0.03 (SEM), *n* = 6]. Cultures with compromised SNBs and more asynchronous firings are associated with low BI values [BI = 0.38 ± 0.08 (SEM), *n* = 3]. After delivering 50 Hz stimuli to the cultures, we noticed opposite outcomes from the two types of cultures (Fig. 3). In cultures dominated by SNBs, networks exhibited LTD in overall change [R = 0.67 ± 0.16 (SEM), *n* = 6; for an example, see Fig. 3*A1–A5*]. The decrease is quantified by a histogram of PSTH area relative changes centered at −20% (for an example, see Fig. 3*A3*). On the contrary, in cultures where SNBs occurred less often with more asynchronous firings in the background, overall LTP was observed [R = 1.73 ± 0.18 (SEM), *n* = 3; for an example, see Fig. 3*B1–B5*]. Figure 3*C*, panels 1, 2, provides examples for potentiated and depotentiated connections and the corresponding change in their CCH, PSTH curves and raster plots. The protocol used in generating the results is CCH – 50-Hz tetanus – CCH.

In a further analysis examining the directionality of these connections, we noticed that the potentiated connections induced by 50-Hz tetanization clustered around postsynaptic neurons (electrodes) rather than presynaptic neurons (grid positions; for an example, see Fig. 3*B1*). In our experiment setup, if the hub is an electrode, it will have only incoming connections; if it is a grid position, it will have only outgoing connections.

**Figure 4.**
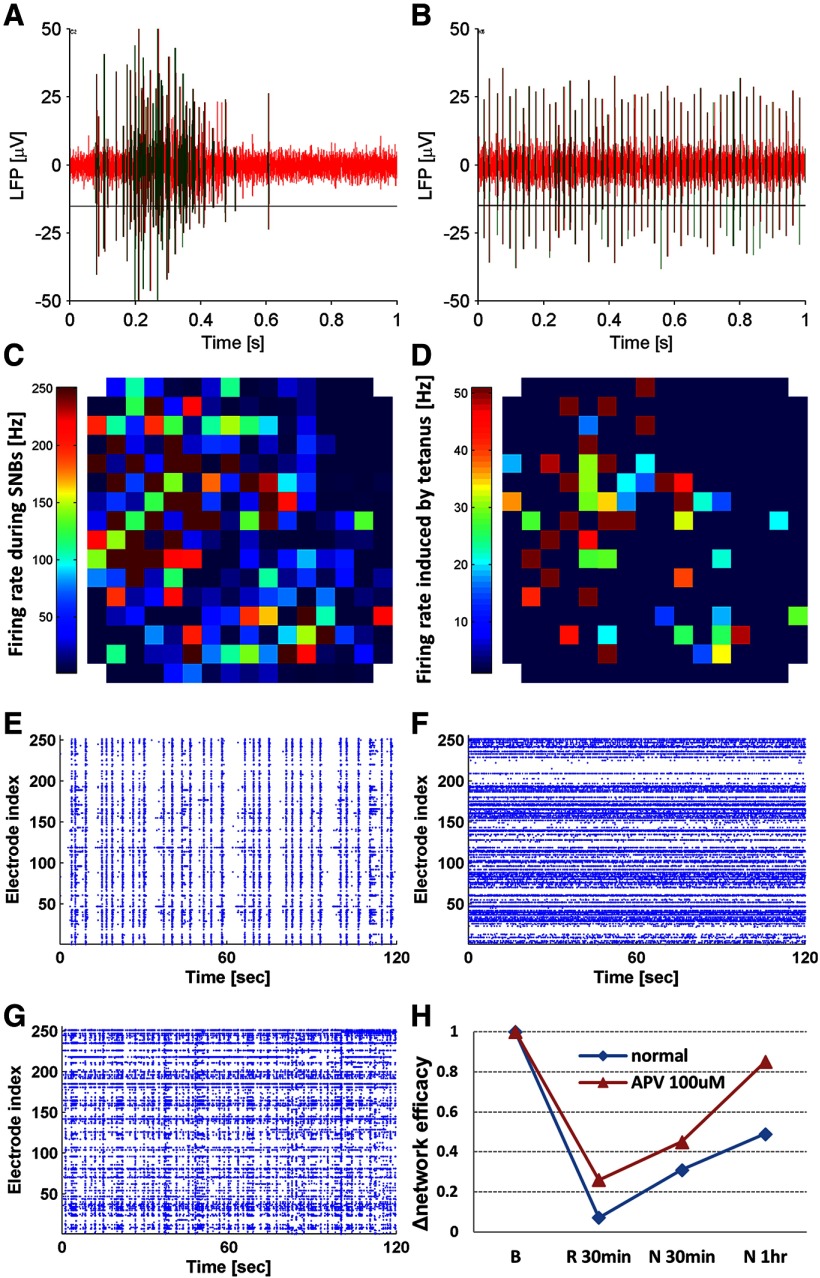
***A–D***, Comparison of network firing rate during SNBs and in response to 50-Hz stimulation. ***A***, Local field potential (LFP) recorded at a single MEA electrode during an SNB. The red trace is noise and the green trace is when LFP exceeds the detection threshold set at −6σ of the noise. ***B***, LFP recorded at a single electrode during 50-Hz light stimulation. ***C***, Network firing rate map during SNBs. Average firing rate was calculated for each electrode over 100-ms time bins during a 5-min recording of network activity with no external input. Firing rate in the top 15% bins was averaged to represent the firing rate during SNBs. No spike sorting was conducted. ***D***, Network firing rate map during response to whole-field 50-Hz tetanization, averaged over 60 trials. Electrode signals collected from multiple neurons were filtered through spike sorting and the signal from the most synchronized neuron was retained for plotting. Notice the difference in color bar scales in ***C***, ***D***. ***E–G***, raster plots of network activity in resting state (***E***), in response to RDMs (***F***), and resting state of a less synchronized culture (***G***). ***H***, Network efficacy change ratio [R = P/|N|; see Materials and Methods, CCH probing, for more details regarding the calculation] from baseline (***B***) to 30 min under RDMs (R 30 min) followed by 30 min in resting state (N 30 min) and 1 h in resting state (N 1 h). APV was added to the culture medium (100 μM) to evaluate the contribution of NMDA receptors.

Comparing the electrode responses to SNBs and 50-Hz tetani, we noticed that spontaneous firing patterns at individual electrodes during an SNB were more concentrated with shorter duration (0.2–0.3×) and larger amplitude (1.5–2×), compared with induced response by 50-Hz stimulation (Fig. 4*A*,*B*). This kind of ultrahigh-frequency spontaneous firing was observed at a large number of electrodes during an SNB (number of electrodes detected at least 50 Hz firing >30; Fig. 4*C*), whereas the number of electrodes detected synchronous firing to 50 Hz was limited (number of electrodes detected at least 50 Hz firing ≤17; Fig. 4*D*).

### RDMs induce LTD prior to learning

Given the analysis above, the question we addressed next is whether SNBs can be suppressed in cultures, while maintaining network viability. Pharmacological and electrical treatments have been proposed on this matter. Pharmacological reagents, such as NMDA receptors antagonist (e.g., APV and Mg^2+^) and gap junction blockers (e.g., carbenoxolone), are able to suppress SNBs to a great extent, but at the same time they also compromise the neuronal network functionality, especially its learning ability. A non-pharmacological method, i.e., randomly applied electrical stimuli through electrodes at high frequency, was suggested to quiet SNBs by mimicking the random noise coming from the external world (Wagenaar et al., 2005). Applying the quieting protocol resulted in enhanced functional plasticity (Madhavan et al., 2006) and increased likelihood of evoked network response (Goel and Buonomano, 2013). We therefore applied optogenetic stimulation in the form of RDMs (see Materials and Methods) to the neuronal networks prior to any learning stimulation, in an attempt to mimicking the effect of sensory inputs on the neocortex in an awake animal. As the cultures were extensively engaged in randomly induced firings during RDMs, SNBs emerged less frequently [BI = 0.86 ± 0.03 (SEM), *n* = 6 (for an example of culture activity before applying RDMs, see Fig. 4*E*); BI = 0.09 ± 0.02 (SEM), *n* = 6; for an example of culture activity during RDM stimulation, see Fig. 4*F*)]. The activity of a less bursting culture seems to fall between the two extreme conditions [BI = 0.38 ± 0.08 (SEM), *n* = 3; for an example of less bursting cultures, see Fig. 4*G*].

**Figure 5.**
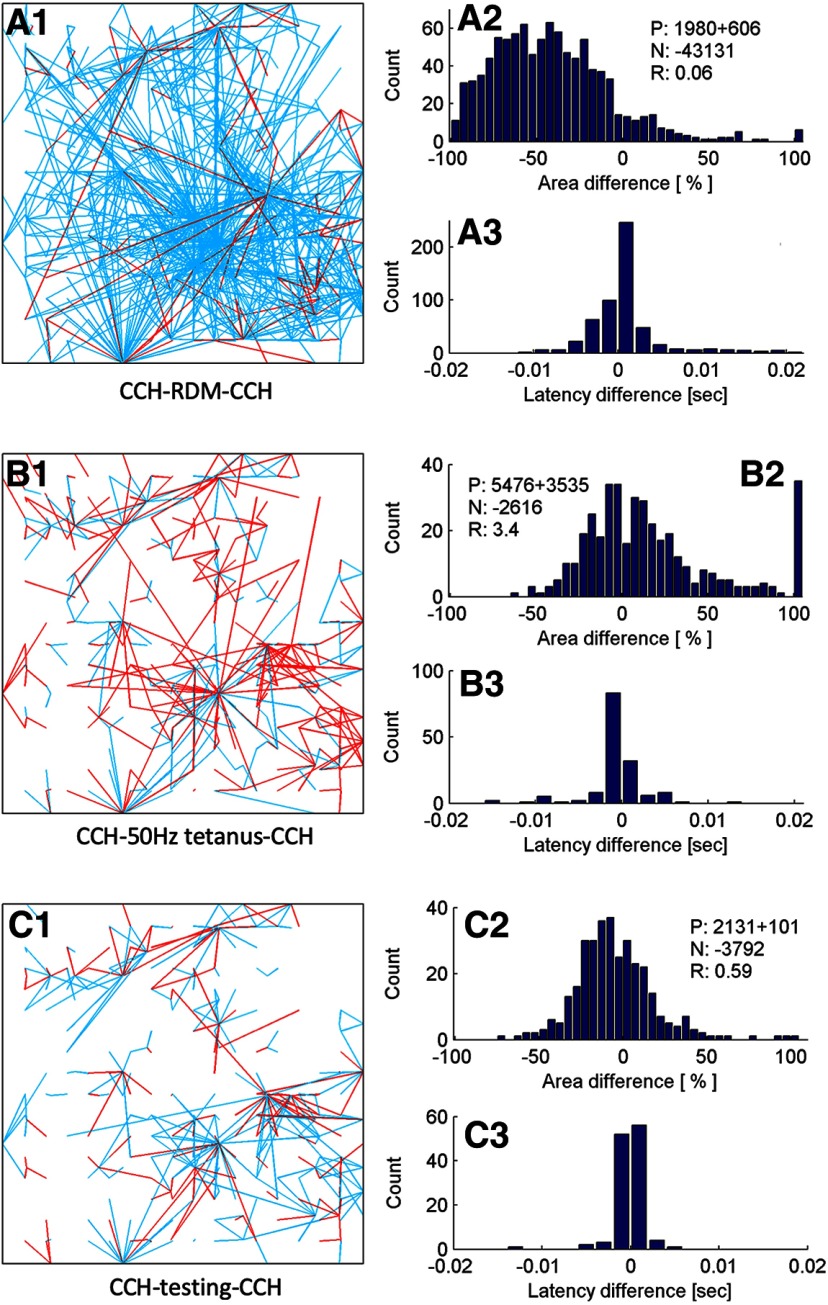
RDM-induced network-wide LTD enables future LTP. ***A1–A3***, Network connectivity changes induced by 30 min RDM stimulation. ***A1***, Potentiated (red) and depotentiated (blue) connections in the culture detected by two CCH probings conducted immediately before and 5 min after the 30-min RDMs. ***A2***, Summary of the relative change in area under the PSTH curves for all the connections detected. See Materials and Methods for the meanings of P, N, and R numbers. ***A3***, Summary of the change in response latency for all the connections detected. ***B1–B3***, Network connectivity changes induced by 50-Hz stimulation conducted after the RDMs (same format as ***A1–A3***). The human face pattern was used as the learning stimulus. ***C1–C3***, Network connectivity changes caused by the testing recording (same format as ***A1–A3***).

We repeated the RDM stimulation protocol in the presence of 100 μM APV in the culture medium to block NMDA receptors and compared the network efficacy changes observed with those seen in control cultures in the absence of APV. A reduced degree of depression immediately after a 30-min RDM and faster recovery after 1 h in resting state were observed when NMDA receptors were blocked (for an example, see Fig. 4*H*). The difference is marked by a network efficacy drop <80% (R = 0.53 ± 0.27, *n* = 2) in APV versus a network efficacy drop >90% (R = 0.07 ± 0.004, *n* = 3) without APV, and faster recovery to the original efficacy at 1 h (R = 1.67 ± 0.82, *n* = 2) after RDMs in APV versus prolonged depression at 1 h without APV (R = 0.36 ± 0.08, *n* = 3). The observed depression is unlikely to be a result of neurons dying, as the cultures survived well for days after the RDM stimulation. The decrease in efficacy in presence of APV is more likely a result of temporary network exhaustion or an NMDA-independent form of LTD (Pöschel and Manahan-Vaughan, 2005). In contrast, the prolonged depression at 1 h after RDM stimulation in the absence of APV, suggests that LTD was induced and the process can be NMDAR dependent. Furthermore, the CCH probings conducted 5 min after RDM stimulation reveals network-wide LTD (R = 0.097 ± 0.015, *n* = 5; for an example, see Fig. 5*A*). RDM-induced network-wide LTD potentially opens a window for enhanced learning, by creating more space for synaptic potentiation. The protocol used in generating the results above is CCH – RDM – CCH.

### Learning specificity emerged after RDM pre-stimulation

**Figure 6.**
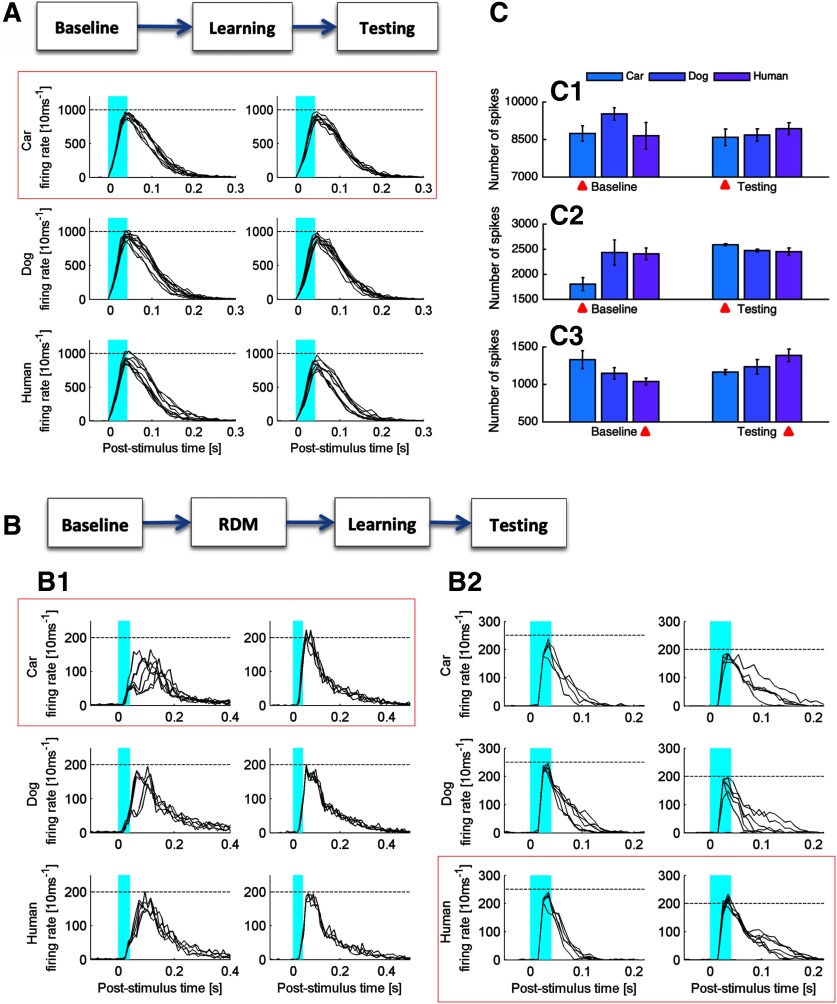
PSTHs of the network responses to the three complex patterns probed at baseline and testing phases. Noisy trials in which SNBs occurred within 1 s before the pattern stimulus onset were removed. Blue indicates the stimulus duration. Red boxes indicate the patterns presented during the learning phase. The left column is baseline response. The right column is testing response. ***A***, Baseline and testing responses recorded from an MEA culture with non-RDM learning. ***B***, Baseline and testing responses recorded from two MEA cultures with learning after RDM pre-stimulation. Dotted lines were drawn at the same heights for all three patterns at baseline/testing to aid visualization of firing rate difference. ***C***, Summarizing the average number of spikes (mean area under PSTH curves + SEM) induced by each pattern over trials during baseline/testing phases for the three cultures shown in ***A***, ***B***. ***C1*** corresponds to ***A***; ***C2*** corresponds to ***B1***; ***C3*** corresponds to ***B2***. Red arrows indicate the patterns presented during the learning phase.

After the cultures were pre-stimulated with RDMs for at least 30 min, we applied the same 50-Hz stimulation protocol. CCH probing results show that, in contrast to the previous LTD outcome in cultures dominated by SNBs, network average efficacy change became LTP (R > 1; Fig. 5*B*), similar to the LTP induced in the SNB-compromised cultures. To further validate that the LTP was indeed induced by the 50-Hz stimulation rather than culture self-recovery from RDM pre-stimulation, we probed network responses to the three complex patterns at the testing phase. As a proper control, network efficacy change caused by the testing (Fig. 5*C*) was not as significant as 50-Hz stimulation, which confirms the minimum disturbance caused in baseline and testing phases. Comparing the PSTH curves of network response to the three patterns at baseline and testing, we found a pattern-specific firing rate increase for the learning stimulus (*n* = 5; for two examples, see Fig. 6*B*). A similar firing rate increase was not observed in the learning experiments without RDM pre-stimulation (*n* = 5; for one example, see Fig. 6*A*). A statistical summary of the network activity over trials during the baseline/testing phases for the three cultures shown in Figure 6*A*,*B* is provided in Figure 6*C*. The protocol used in generating the results above is CCH – RDM – CCH – 50-Hz tetanus – CCH.

**Figure 7.**
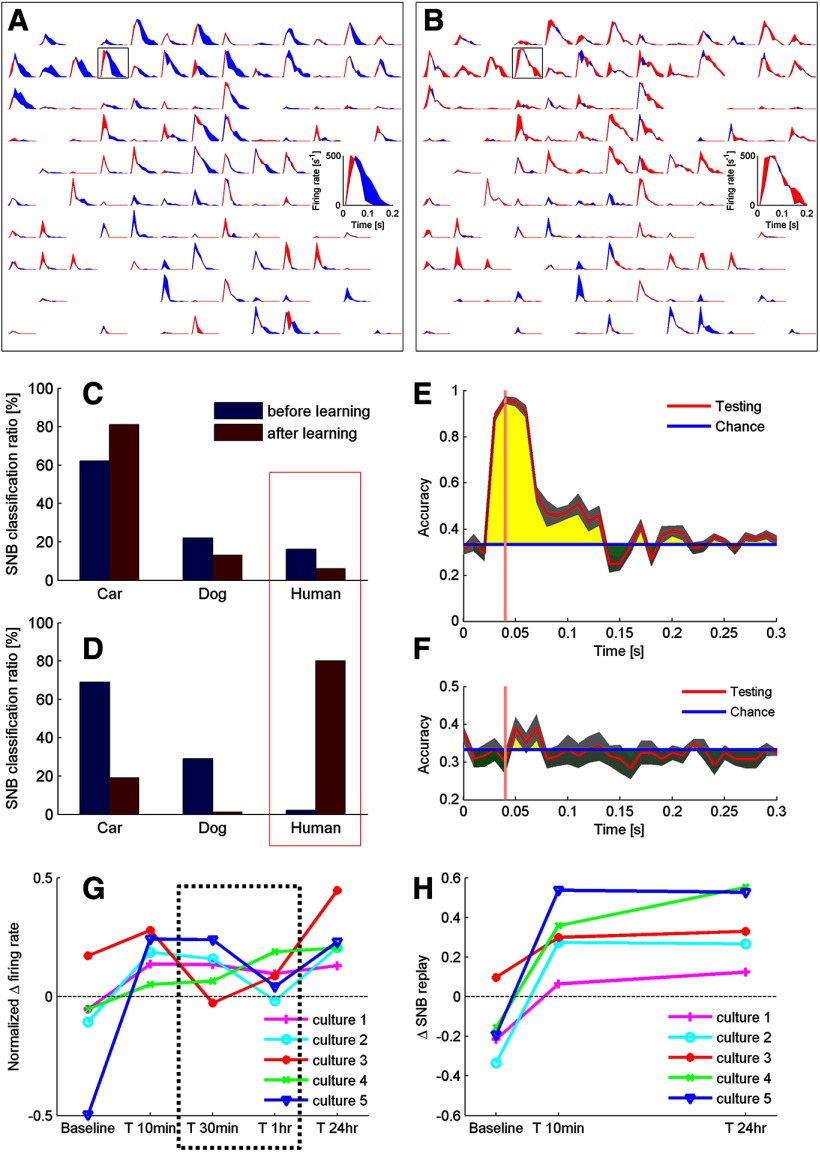
***A***, ***B***, PSTH difference in response to the training and control patterns at individual electrodes. ***A***, Difference between the responses to a human face (trained) and a car front (control) stimulus pattern at baseline. Red indicates a higher PSTH to the human face and blue indicates a higher PSTH to the car front. PSTHs are summed over 20-ms bins. An average PSTH curve of 10 trials is calculated to represent the electrode response to the probing pattern. The boxed electrode response is enlarged as the inset; *x*-scale is [0, 0.2 s] for all; *y*-scale is [0, 500] spikes/s for all. Only the most active portion of the electrode array is shown (10 × 13 electrodes). Only the response to car front is shown as the response to a control pattern for clarity. ***B***, Difference between the responses to a human face (trained) and a car front (control) stimulus pattern at testing (same format as ***A***). ***C***, ***D***, SVM classified labels for SNBs recorded before (blue) and after learning (red) in the conventional learning paradigm (***C***) and the RDM-learning paradigm (***D***). Five-minute continuous recordings of network spontaneous activity were used. Red box indicates the trained pattern. ***E***, ***F***, SVM classification accuracy on pattern-induced responses (***E***) and after we randomized the pattern labels for training as a control (***F***). The pink bar at 0.04 s indicates the termination of light stimulus. Gray shading indicates the SEM of 10 trained SVMs. Yellow indicates classification accuracy above chance level and green for below chance level. ***G***, ***H***, Memory consolidation in cultures with extended testing phase. ***G***, Network response to the trained pattern was probed at 10 min, 30 min, 1 h, and 24 h after the learning phase. Network firing rate induced by the image cues was used to evaluate memory recall. Firing rate discrepancies between the familiar pattern and the two control patterns were averaged and then the value was normalized with respect to the familiar pattern to make the results comparable among cultures (see Materials and Methods, Evaluation metric, for more details regarding the calculation). ***H***, SVM classification of SNBs recorded at baseline, 10 min and 24 h after learning was conducted by using the network responses obtained in ***G*** as training sets. SNB ratios (see ***C***, ***D*** for an example) between the familiar pattern and the two control patterns were averaged to make the results comparable among cultures (see Materials and Methods, Evaluation metric, for more details regarding the calculation). Same cultures as in ***G***.

The pattern-specific firing rate increase indicates that the neuronal networks have acquired familiarity to the trained pattern. Therefore, we conclude that learning after RDMs is more efficient than learning without RDM pre-stimulation. The ability to recognize familiar patterns seems to be an emergent and intrinsic network property, in which information of the complex patterns is separated in a high-dimensional feature space. PSTH curves at individual electrode channels evoked by the three patterns were compared before and after learning (Fig. 7*A*,*B*). Firing rate increase to the familiar pattern was observed at many channels widely distributed in the network.

The experimental paradigm was therefore updated from “baseline → learning → testing” (protocol is CCH – baseline – CCH – 50-Hz tetanus – CCH – testing – CCH, referred as the conventional learning paradigm from here on) to “baseline → RDM → learning → testing” (protocol is CCH – baseline – CCH – RDM – CCH – 50 Hz – CCH – testing – CCH, referred as the RDM-learning paradigm from here on). Baseline recording was conducted before RDM pre-stimulation to prevent unwanted network recovery induced by the baseline recording. This way, the network baseline responses were generally higher than the testing responses (Fig. 6*B2*), because RDMs induced significant network LTD, and the subsequent learning only selectively potentiated a subset of the connections.

### Memory consolidation *in vitro*

What we have observed so far is at best early-phase LTP (Huang, 1998; Blundon and Zakharenko, 2008). During the experiments, CCH probings were conducted 5 min after learning, and network responses to the three patterns were obtained 10 min after learning at the testing phase. This time scale is longer than short-term memory but still short for long-term memory. Given that SNBs occurred sporadically in the background, which might result in an ongoing modification of network connectivity, we wanted to address the question whether the encoded familiarity would be consolidated into long-term memory.

In intact animals, memory consolidation has been correlated with high-frequency oscillations that can be observed during sleep. Cultures with SNBs have been proposed as a slow-wave sleep model (Colombi et al., 2016). Therefore, it is reasonable to hypothesize that SNBs may be retaining the encoded pattern by spontaneously initiating neuronal activities through the potentiated connections. In order to decode the information contained in the SNBs, we used an SVM approach to classify SNBs.

At first, SVM classifiers were trained to recognize network responses induced by the three complex patterns. Then, the SVM classifier trained with pattern-induced responses obtained at the baseline phase was applied to classify SNBs in the 5-min continuous recording obtained before the baseline phase; the SVM classifier trained with pattern-induced responses obtained at the testing phase was applied to classify SNBs in the 5-min continuous recording obtained immediately after the learning phase. Classified labels of SNBs before and after learning are compared. The results show that after the RDM-learning paradigm, a larger portion of SNBs were classified as the trained pattern (Fig. 7*D*), indicating a higher similarity in the network activity pattern. In other words, SNBs carried more information of the trained pattern after learning. Therefore, the trained pattern is likely being replayed during SNBs, potentially undergoing memory consolidation. On the contrary, SNBs after the conventional learning paradigm did not resemble the trained pattern more (Fig. 7*C*). Separately, we validated the trained SVM classifiers by looking at their classification accuracy of an independent set of pattern-induced network responses (60% data were used for training, 40% data for testing) recorded at baseline. The results show good accuracy at 30–120 ms, while the pattern stimuli were delivered at 0–40 ms (Fig. 7*E*). When the pattern labels were randomized prior to SVM training, the classification accuracy reduced to chance level (Fig. 7*F*).

To further investigate the long-term retention of the encoded familiarity, we extended the testing phase in an independent set of experiments by including three sessions of testing spaced at 30 min, 1 h, and 24 h after the learning phase in addition to the original testing conducted at 10 min. While network firing rate distinction was observed to be not well maintained 30 min after learning, network firing rate to the familiar pattern re-appeared to be on top when probed at 24 h after learning (Fig. 7*G*). A similar effect has been reported by another group (Chiappalone et al., 2008, their Fig. S1), who found network response maintenance 1 d after LTP induction experiments. So, if we only focus on the familiarity recall with 1-d retention (24 h), the network firing rate discrepancy between familiar and control patterns is as good as when recalled immediately after learning (10 min), implying the existence of memory consolidation. A similar trend was observed with the corresponding SNB replay ratio (Fig. 7*H*). The protocol used in generating the results above is CCH – RDM – 50-Hz tetanus – CCH (10 min) – CCH (30 min) – CCH (1 h) – CCH (24 h).

### Comparison between conventional learning and RDM-learning paradigms

**Figure 8.**
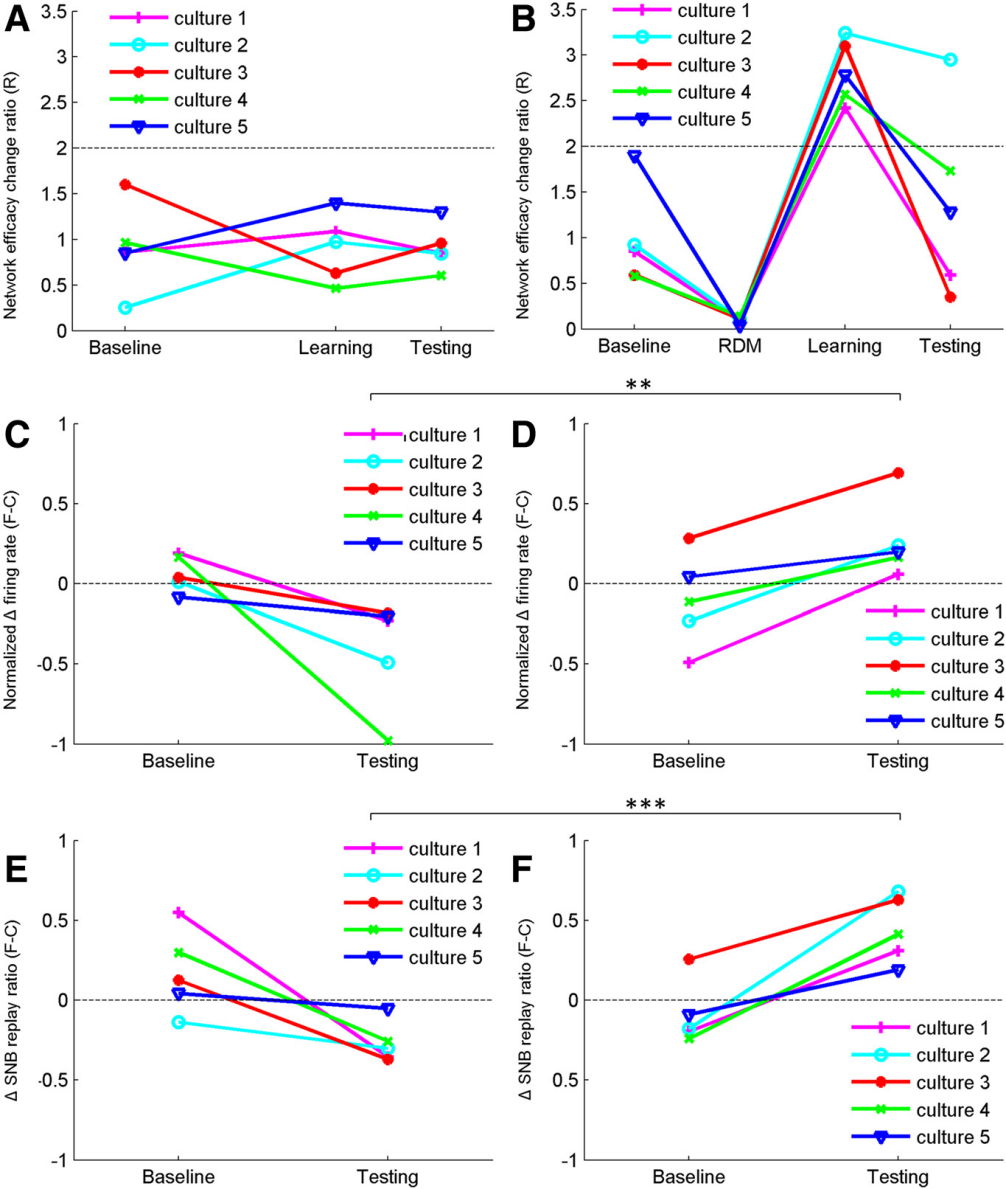
Summary of culture performance under the conventional learning paradigm (***A***, ***C***, ***E***) and the RDM-learning paradigm (***B***, ***D***, ***F***). ***A***, ***B***, Network efficacy change ratio [R = P/|N|; see Materials and Methods, CCH probing, for more details regarding the calculation] induced at the baseline phase, RDM pre-stimulation, learning phase, and testing phase for experiments conducted under the conventional learning paradigm (***A***) and the novel RDM-learning paradigm (***B***). ***C***, ***D***, Network firing rate discrepancy between familiar and control patterns (see Materials and Methods, Evaluation metric, for more details regarding the calculation; *p* < 0.01 (**) for unpaired two-sample *t* test) for experiments conducted under the conventional learning paradigm (***C***) and the novel RDM-learning paradigm (***D***). ***E***, ***F***, Change in SNB replay ratio for the familiar pattern (see Materials and Methods, Evaluation metric, for more details regarding the calculation; *p* < 0.001 (***) for unpaired two-sample *t* test) for experiments conducted under the conventional learning paradigm (***E***) and the novel RDM-learning paradigm (***F***). Different sets of cultures were used in each panel.

Summarizing all the findings above, we present here the major difference between the conventional learning paradigm and the novel RDM-learning paradigm. As the dynamics of *in vitro* dissociated neuronal networks are highly variable and noisy in nature, we have had plenty of outlier cultures with unique behavior that could not be subsequently reproduced. This paper therefore focuses on the most reproducible observations. When 50-Hz tetanus was given to cultures during the learning phase, little LTP (R < 2) was observed following the conventional learning paradigm (Fig. 8*A*), whereas significant LTP (R > 2) was observed following the RDM-learning paradigm (Fig. 8*B*). One prerequisite for the training to be efficient in the RDM-learning paradigm was that the RDM pre-stimulation had to induce major depression in the network efficacy [R = 0.097 ± 0.015 (SEM), *n* = 5; Fig. 8*B*]. In cultures that RDM pre-stimulation did not induce sufficient depression, the subsequent firing rate distinction was compromised. In the conventional learning paradigm, network firing rate to the trained pattern was decreased in general (Fig. 8*C*), suggesting mild LTD induction following the 50-Hz tetanization. Although pattern-specific LTD might be an alternative learning outcome, the encoded LTD is unlikely to be replayed by the background SNBs (Fig. 8*E*) and therefore will eventually be lost over time. In the RDM-learning paradigm, network response to the three patterns was first greatly suppressed by the RDMs and then a subset of neurons was selectively stimulated and recruited to store the encoded familiarity, resulting in a comparatively higher network firing rate to the trained pattern among the three (Fig. 8*D*). More importantly, postencoding SNBs spontaneously reactivate the memory engram (Fig. 8*F*), which provides means to maintain the strengthened pathways in the network and potentially creates a robust model for learning and memory studies *in vitro*.

## Discussion

The goal of the experiments described above was to study learning and memory in neuronal networks. A major issue holding back progress in networks formed by cultured cortical neurons is the spontaneous occurrence of highly SNBs.

### Development of SNBs

SNB activity patterns change with the developmental stage of cultured cortical neurons, and they have been shown to disrupt the encoding of short-term memory (Dranias et al., 2015; Ju et al., 2015). In addition, it has been postulated that SNBs are essential for neuronal network development and maturation (Luhmann et al., 2016). The fact that properly maintained MEA cultures can survive for months (Potter and DeMarse, 2001) creates an opportunity to study the development of the functional activity of these neuronal networks in detail. In our experiments, SNBs emerged as early as DIV8, indicating the existence of global connections at this early age, However, long-range connections were not detectable by our CCH analysis until the culture had adequately matured. The sparsity of detectable global connections before DIV22 is likely the result of synaptic efficacies being relatively weak initially. Normally, spatial and temporal summation of multiple inputs is required to trigger a postsynaptic action potential. In order to be detected by CCH probing, a global connection must be strong enough to trigger the firing of the postsynaptic neuron by itself. The long-range connections that were uncovered in the connectivity map at DIV19 (Fig. 2*E*) by lowering the CCH detection threshold possibly represent newly formed connections which are still relatively weak. Some of these can strengthen as the network matures. In other words, the CCH analysis relies on strong connections to reconstruct the network architecture. We have focused on such strong connections, because they are more stable and reliable when we look for subtle changes due to plastic modification.

### Long-term effect of SNBs

In cultures dominated by SNBs, high frequency stimulation with a training pattern caused an LTD of network activity (Fig. 3*A1–A5*). The overall LTD is consistent with observations reported earlier (Chiappalone et al., 2008), that high-frequency tetanization caused an overall decrease in evoked network response, rather than a global increase as conventionally expected. For cultures in which SNBs were sparse and which displayed more asynchronous firing, tetanization with a pattern resulted in LTP (Fig. 3*B1–B5*). Potentiated connections clustered around a few postsynaptic neurons (Fig. 3*B1*). This result supports the idea that these postsynaptic hub neurons, receiving multiple inputs from concurrently stimulated dot positions of the training pattern, have a higher chance to fire synchronously in response to high-frequency stimuli, which in turn results in potentiation of the synapses between them and their presynaptic inputs following the “Hebbian” rule.

An individual MEA electrode may record from between one and four neurons (Litke et al., 2004). Therefore, network firing frequency recorded by the MEA during SNBs can be much higher than the 50-Hz frequency we used for tetanization. It is reasonable to assume that the neuronal networks have been excessively potentiated by SNBs, leaving little space for future LTP. In other words, SNBs impose a higher LTP threshold for *in vitro* neuronal networks.

SNBs have been hypothesized to assist the synaptic development and maturation of the neuronal networks in young cultures (Kerschensteiner, 2014). However, the results described above potentially implicate the adverse long-term effect of having SNBs in cultures.

### Learning familiar inputs after RDMs

We have shown that stimulating the networks with RMDs induces a form of LTD, in which the frequency of SNBs is substantially reduced. We have used a BI to monitor this process. If we view the baseline network activity with abundant SNBs as purely ordered (for an example, see Fig. 4*E*) and activity during RDMs as purely disordered (for an example, see Fig. 4*F*), then the activity of the networks after RMD treatment (for an example, see Fig. 4*G*) seems to fall at the “edge of chaos,” which has been predicted to have optimal computational power (Bertschinger and Natschläger, 2004; Legenstein and Maass, 2007).

High-frequency tetanization after RDM pre-stimulation induced pattern-specific LTP. The pattern-specific network firing rate increase indicates that the neuronal networks have acquired familiarity to the trained pattern. Therefore, we conclude that the ability to recognize familiar complex patterns is an emergent and intrinsic property of neuronal networks that are maintained *in vitro*. The network’s ability to discern patterns and recognize a familiar input has been predicted by previous studies with simulated neural network models. Constrained by the spatial resolution of MEAs, we could not measure the activity of each individual neuron in the network, and therefore we could not identify the critical neurons and subnetworks responsible for familiarity, as was done for the simulated neural networks. Nevertheless, based on the PSTH curves of network responses at individual electrodes, we can tell that the firing rate increase to the familiar pattern is distributed to many channels in the network, implying it reflects a network property. Similar observation has been found in reservoir computing (Subramoney et al., 2019).

In the learning experiments, after we applied pattern-specific optogenetic stimulation, we probed the network responses to both trained and control patterns. By including control patterns, it came to our attention that in cultures dominated by periodic SNBs, high-frequency tetanization induced little learning specificity. It was only after we pre-stimulated the cultures with RDMs which counteracted the SNBs by reducing network efficacy on average, we started seeing a difference in evoked network responses induced by high-frequency tetanization between the training and control patterns (Fig. 6). By showing that the network response change is directly associated with the trained pattern, we established a more specific causal relationship that is less likely to be confounded by fluctuations in network background activity. Missing proper controls and the lack of direct causality in the early studies prior to Wagenaar et al. (2006b) potentially undermine their reliability, because changes in the network firing rate, SNB frequency or response latency are not exclusive to learning but could simply be caused by fluctuations and drifts in network excitability.

The flexibility provided by optogenetic stimulation and the novel CCH probing method allows us to examine changes in the network at each step and provides more insight into the functional role of SNBs. For cultures without RDM pre-stimulation, high-frequency tetanization induced LTD. Whereas for cultures after RDM pre-stimulation or cultures with naturally compromised SNBs, high-frequency tetanization induced LTP. Applying CCH probings, we discovered that RDM pre-stimulation induces a network-wide depression. These phenomena together suggest chronic SNBs have excessively potentiated the networks, leaving little space for future potentiation (Wagenaar et al., 2006b). Therefore, when high frequency tetani were delivered, the networks underwent changes skewed to the LTD side, impairing the learning specificity. We suspect that the equivocal observations from previous MEA studies stem from differences in network SNB levels at the resting state. Many of the aforementioned studies did not characterize the bursting status of their cultures, leaving the problem open for further investigation.

For experiments performed using the RMD-learning pathway, the network baseline responses were generally higher than the testing responses (Fig. 6*B2*), because RDMs induced significant network LTD, and the subsequent learning only selectively potentiated a subset of the connections. Nevertheless, occasionally we observed the opposite, namely that the testing responses were higher in firing rate than baseline responses (Fig. 6*B1*). This occurred when there was an increase in average IBI duration for SNBs in the background from baseline to testing, suggesting that the networks were more engaged in SNBs at baseline, but more entrained to external stimuli at testing. This phenomenon can happen in either paradigm, i.e., learning with or without RDM pre-stimulation. When it occurred in the conventional learning paradigm, despite the overall firing rate increase in evoked network responses at the testing phase, no learning specificity to the trained pattern was co-observed, indicating the change was not correlated with familiarity encoding. This phenomenon is easily confused with a real learning effect when there is no rigorous control.

Based on our experience, a sufficiently dense, viable MEA culture will be dominated by SNBs from DIV13 onwards. Although the bursting has been regarded as seizure-like in some studies (Wagenaar et al., 2005; Ahn et al., 2017), it is more likely to be a result of the absence of external inputs during the development of neuronal networks (Madhavan et al., 2006). The frequency of SNBs in a mature culture stabilizes at 0.1–0.2 Hz, which falls into the frequency range of the δ brain waves detected by EEGs (0.1–4 Hz). δ Waves are characterized as the slowest brain waves with the highest amplitude and are commonly found in stage N3 slow-wave sleep (Vlahou et al., 2014). Therefore, a few recent studies suggest that the emergence of SNBs signifies MEA cultures in a state equivalent to chronic sleep. Similarity in gene expression profiles between cortical cultures and sleep models provides further support for this notion (Hinard et al., 2012). Carbachol, a cholinergic agonist, was applied to the cultures and successfully transformed the network activity from resembling slow-wave sleep to more like activity during rapid eye movement (REM) sleep (Colombi et al., 2016). We noticed that the network activity induced by RDM pre-stimulation (Fig. 4*F*) showed a similar pattern to the Carbachol-induced REM-like activity. The fact that RDM pre-stimulation counteracted SNBs and caused network-wide depression may be suggesting how synaptic efficacy homeostasis is established in the brain.

A few studies investigating learning and memory in cultured neuronal networks attempted to circumvent the SNB obstacles. One group was able to show the networks’ ability for pattern separation with L-shape patterns, but they argued that electrical tetanization with a frequency above 200 Hz was required to observe the learning specificity (Ruaro et al., 2005). It seems that using a higher frequency may be able to conquer the over-potentiation problem caused by SNBs. Nevertheless, inducing such an ultrahigh-frequency tetanus is not feasible with our optogenetic stimulation. The membrane properties of cortical neurons prevent them from firing synchronously faster than 50 Hz. Therefore, it is impossible to induce 200-Hz spiking activity in a physiological condition. Another group demonstrated learning specificity in networks’ direct response to stimuli after pharmacologically inducing neurogenesis (Tanaka et al., 2017), which potentially introduced more immature synapses to the network whose efficacy had not yet been over-potentiated by SNBs. In a study that failed to induce network LTP with high-frequency tetanization (Chiappalone et al., 2008), it was shown that associative memories could be induced in the cultured networks by pairing a session of high-frequency tetanus with an in-phase single pulse applied at a spatially distant electrode. The mechanism underlying the in-phase pairing protocol for associative memory induction was not explicitly explained. In a following review article, it was speculated that the in-phase pairing protocol induced stronger network bursts than a session of high-frequency tetanus alone (Bologna et al., 2010). In addition, metaplasticity has been reported underlying associative memory (Sharma and Sajikumar, 2014; Xu et al., 2014), and the in-phase pairing protocol might be an efficient way of modifying metaplasticity in the cultured networks, which in turn modifies the threshold for synaptic plasticity induction.

SNBs re-appear after the RDM-learning-testing sequence, and 24 h later they still encode information specific to the learned pattern (Fig. 7), suggesting that they take part in memory consolidation. Regarding why the network firing rate to the familiar pattern was not consistently the highest for the shorter retentions (30 min and 1 h), we do not have an explicit answer for now. It has been shown that postencoding maintenance of the activity pattern present during learning is critical for memory consolidation (Guzman-Ramos and Bermudez-Rattoni, 2011; Park et al., 2016). Specifically, a time course study that investigated the influence of transient inactivation of perirhinal cortex on recognition memory (Winters and Bussey, 2005) showed that memory consolidation was impaired when the cortical neuron activity was suppressed by lidocaine shortly after encoding (0–20 min), but no impairment was found if the suppression was incurred 40 min after encoding. It was then suggested that at some time point beyond 20 min after encoding, the memory trace became sufficiently strengthened to resist any disruption to the neuronal activity. On account of this, it is possible for us to observe the firing rate decay at 30-min and 1-h time points, while the memory was still consolidated at 24 h. On the other hand, the weakened response to the familiar pattern at 30 min and 1 h might be a network strategy to get ready for new information processing (Oliveira et al., 2010) and prevent encoded memory being overwritten. Nevertheless, it is important to note that each of the cultures that we studied is a single network, which carries no hierarchical regulations like in the brain. We should take caution to avoid over-explaining the firing rate decay, as it may be simply due to network exhaustion after multiple testing sessions in a relatively short window.

The presence of SNBs may not be entirely a bad thing. In fact, spontaneous synchronized activity is common to the brain at many levels (Corlew et al., 2004; Blankenship and Feller, 2010). On the largest scale, spontaneous oscillations have been observed for the entire brain by fMRI and EEG (Burke et al., 2013). On the brain circuit scale, it has been shown that high-frequency synchronized activity in the hippocampal-cortical dialogue during slow-wave sleep is involved in memory consolidation (Mitra et al., 2016), reflected as sharp-wave ripples in hippocampus and sleep spindles/k-complexes in cortex (McVea et al., 2016). Neuronal activity patterns evoked during learning have been found to be replayed in the hippocampus during sleep (Breton and Robertson, 2013). There is a chance that SNBs *in vitro* function in a similar way to enforce the strengthened connections. The SVM classification results show that the encoded pattern was “replayed” by SNBs after learning. It sheds some light on the existence of memory consolidation mechanism in neuronal networks. Therefore, even in the presence of SNBs, *in vitro* neuronal networks can be used to study learning and memory with proper preparations.

## Conclusion

In this study, a novel probing method was applied to scrutinize the network change in response to learning. By counteracting spontaneously occurring SNBs, we managed to induce pattern-specific familiarity, an observation that is comparable to what has been demonstrated *in silico* and has never been reported before for *in vitro* neuronal networks. The postlearning activity pattern of SNBs carried more information of the familiar stimulus, suggesting a mechanism of memory consolidation. It is concluded that *in vitro* neuronal networks can acquire familiarity to complex patterns with specificity, and we suggest a novel elucidation to the role of SNBs as a double-edged sword that disrupts the encoding of new memories and facilitates the consolidation of existing memories at the same time.

## Synthesis

Reviewing Editor: Juan Burrone, King's College London Decisions are customarily a result of the Reviewing Editor and the peer reviewers coming together and discussing their recommendations until a consensus is reached. When revisions are invited, a fact-based synthesis statement explaining their decision and outlining what is needed to prepare a revision will be listed below.

This is a much-improved manuscript that now shows important insights into memory, recognition and familiarity of patterns in in vitro networks. This version of the manuscript has significantly improved in clarity and provides more detailed description of the methods and results. However, the last section of this paper, which comprises figures 6 to 8, is less clear and requires more information. Figure 6, especially, appears to show some contradictory results.

Below are main points raised by the reviewers:

Fig 5B1 shows a general potentiation in the protocol CCH-learning-CCH. So why is there no potentiation when the same protocol, baseline-learning-baseline, is used?

Also, when looking at fig 6C1 an overall drastic increase in the response to the "dog" is observed.

It seems there are fluctuations in the responses to the three stimuli (car, dog and human) which are hard to explain, and they have the same magnitude as the changes claimed as "learning". Are they just normal noise and fluctuation of the responses?

The same applies to Fig. 6C3. The response to the stimuli in baseline and after learning are big, the "car" response clearly decreases, the "dog" is stable, the learnt "human" increases. The change of the learnt vs non-learnt are very similar. Where does this variability arise? This is an important point as it can affect the conclusions reached.

In addition, the trend described above in fig 6C3, is not clear in fig. 6B2, which relates to it. The baselines seem to be equal for the 3 stimuli, although in fig. 6C3 it is clearly not equal. Where does this apparent difference arise? In fig. 6B2 right panels, the dotted line is lower, although I would not expect this when looking at fig. 6C3. Finally, the normalizations shown in fig 7 and 8 which summarize the all "learning& memory" study are not clear. It is therefore important to provide a detailed methodology that explains the methods and calculations carried out to obtain the different plots shown in fig. 6, 7 and 8.

Minor comments

In the MS the protocols are always presented at the end of the presentation of the results.

However, there are so many protocols, and it is not always clear, given a single culture, how they are temporally organized within the experiments.

METHODS

The lambda of the Poisson process, was it calculated in baseline condition of spontanoues activity? In a time bin of which size? The CCH esseniatlly caculated a PSTH in bins of 30 ms (the duration of the ON stimulus), but with overalapping sliding windows shifted of 1 ms. So the lambda should also be calculated in a 30 ms time bins.

In the section "culture exclusion", at line 222 network efficacy is mentioned, but never defined before in the Methods. All analysis methods have to be explained clearly in the Methods section. Especially the analysis methods used for fig 6, 7 and 8 where normalization is fundamental to assess the solidity of the results.

At line 229/230, it is written "three cultures for the RDM-learning paradigm and five cultures for the RDM-learning paradigm," it looks it is the same written twice. Please correct and verify numbers.

RESULTS

Between line 344 and 349, it is written that the LTD can be NMDA-independent and later the opposite is written. Please check this and explain more clearly why it is postulated as an NMDA dependent process, even though results with APV tend to show the opposite.

Fig. 4 and 5 show one culture results, please present also statistics over all experiments (I could not find it and f it is presented in a different place, it would be better to present it when fig 4 and 5 are discussed).

LEGENDS

Fig. 3: the calculation to obtain P and N are described, please put all methodologies in a devoted clear section of the Methods section. Also, P and N look not just the sum, but an integral which takes into account the bin size. Is it the case? Because the sum cannot give the values reported. Please explain. Also what is the size of the bins in the PSTH presented? As I asid the CCH is a sliding time windows PSTH with bin of 30 ms. So if you used bins of 30 ms for the PSTH you should get similar profiles and amplitude of the CCHs.

## Author Response

Synthesis of Reviews:

Synthesis Statement for Author (Required): The paper shows novel data that could have potentially important impact on learning and memory in in vitro networks. It examines learning and memory in dissociated cultures, where ChR2-expressing neurons grown on MEAs are trained with one of three optical patterns. Results show that the network firing rates to evoked patterns are larger in response to the trained ("familiar") pattern compared to the untrained (novel) pattern. Particularly interesting is the use of RDMs to quieten SNBs, allowing the network to be trained to a particular input pattern. In addition to this example of in vitro learning the authors observe a "replay" or "consolidation"-like effect in which the information available in the spontaneous patterns of network activity is richer for the familiar pattern (i.e., using an SVM it was possible to determine which training stimulus had been presented). However, the manuscript lacked methodological detail, especially regarding the analysis and statistics used, which was an important issue when interpreting the data. The manuscript was also found to be confusing in parts and should be carefully re-written to better explain the findings presented here. This includes having a detailed Methods section that clearly explains the protocols employed and a Results section that allows a clear evaluation of the data without ambiguity. Below are a list of issues raised by the reviewers that the authors need to address.

In general, methods, results and discussions are mixed in the manuscript, complicating any clear evaluation of the results.

Q1. There is very little statistics in the paper, this is perhaps because most of the results from the individual experiments are presented. Nevertheless, statistical analyses are needed. Some of the key results are presented in figure 8. Are the 5 cultures shown in each group (Conventional x RDM) all the cultures tested, or was there some inclusion/exclusion criteria? Is there a statistical difference between testing between both groups for the three measures (delta efficacy, delta firing rate, and delta SNB)?

We tested more cultures than the five presented for each group. Before each experiment, we recorded a 10-min session of the culture's activity at rest and excluded cultures with frequency of background SNBs more than 0.2 Hz (too active) or less than 0.05Hz (too quiet). Cultures whose overall firing rates changed significantly before and after the baseline recording phase were also excluded, because they were found to be unstable and produce inconsistent results for the subsequent phases. For the RDM learning paradigm, cultures whose bursting activity was not significantly suppressed by the RDMs (R > 0.2) were excluded from the study, because their subsequent learning potential was compromised. Eventually we were left with between 6 and 10 cultures for each group/experiment, and we have shown the five most representative cultures.

In terms of the statistical difference in the testing results between the two groups, it is most meaningful to look at the measures of delta firing rate and delta SNB replay. Delta efficacy should not change much before and after testing because conservative settings (three patterns each were presented 10 times in random

2

orders with 10 sec intervals and 100 msec stimulation time) were used for baseline and testing recordings to avoid inducing any LTP/LTD effect outside the period of learning stimulation. The larger change in efficacy for testing after the RDM-learning paradigm is most likely to result from the extended learning window that followed the RDMs.

We applied the unpaired two-sample t-test to our data, assuming equal sample variance. The pooled standard deviation of the two samples is used as an estimator of the common variance given the same sample size.

𝑡 =

𝑋 ®1 − 𝑋 ®2 𝑆𝑝 √2 𝑛

where

𝑆𝑝 = √

𝑆𝑋1 2 + 𝑆𝑋2 2 2

Here 𝑆𝑋1 2 and 𝑆𝑋2 2 are the unbiased estimators of the variances of the two samples (X1 for the conventional learning paradigm, X2 for the RDM-learning paradigm). 𝑆𝑝 is the pooled standard deviation for n = n1 = n2. The degrees of freedom for this test is 2n − 2 where n is the number of participants in each group (n = 5).

Please refer to the table below for the statistical testing results. The p-values have been added to Figure 8 accordingly.

𝑋 ®1 𝑋 ®2 𝑆𝑝 |t| df p-value (two-tailed) Normalized △ firing rate -0.42 0.27 0.29 3.76 8 0.01 △ SNB replay ratio -0.26 0.44 0.17 6.52 8 0.001

If we set the p-value threshold as 0.01, the null hypothesis will be rejected for both statistical tests, and we accept the alternative hypothesis that there is a statistical difference between the two groups in terms of delta firing rate and delta SNB replay ratio.

Q2. Was the chosen training stimulus (car, dog, human) chose at random? It looks like the average valued of 7G Baseline is below 0.

Yes, the training stimulus was chosen at random. As the data analysis was done after the experiment, it was impossible for us to know the rank order of the three patterns in network firing rate induction before applying the training stimulus.

That the majority of cultures have a baseline below 0 in Figure 7G is a chance event. As this experiment was designed and conducted towards the end of the project, only six cultures were tested and four out of the six have negative baselines. We

3

excluded one culture because of an outlier at T=1hr. The figure below shows the single culture that excluded as a dotted black line.

Q3. Was the SVM performance on the evoked image discrimination reported? Or was that not done because it was not possible to cross-validate (e.g., leave-oneout)?

The SVM performance on the evoked image discrimination was reported. We evaluated the SVM classification accuracy on the training stimuli by using a 60/40 split for training/testing. Please see the Methods section where it says that, "60% of the pattern-induced network responses were used to train the SVM classifier and the remaining data was classified by the trained classifier" for validation purposes. In Figure 7E, the classification results on the test set show good accuracy at 30-120 msec post stimulus delivery. To eliminate the possibility of other factors causing the high classification accuracy, we also created train/test sets in which the training stimulus labels were shuffled and then we observed that the classification accuracy reduced to chance level (Figure 7F).

We hope the results described above answer the question raised by the reviewer(s). It is possible to do cross-validation with the data, but because the test accuracy is good enough with a 60/40 split of train/test sets, we think it is not necessary to use leave-one-out cross validation.

Q4. The presentation can be a bit confusing. E.g., the switch between delta-efficacy and P/N terminology. Also, the figures axes should be labeled with "delta ... (Familiar-Novel)" to help the readers understand if the delta is between stimulus classes rather than time points.

This is a good point. "△ network efficacy" in Figure 8AB is the difference in networkwide efficacy between two time points, which is essentially the same as the P/|N| ratio. We decide to change the y-axis label for Figure 8AB to "Network efficacy change ratio (R)" to be consistent with the R (R = P/|N|) used in Figure 3 and 5. For Figure 7GH and Figure 8CDEF, "normalized △ firing rate" and "△ SNB replay" involves the comparison between the stimulus classes. We decide to rename them to "normalized △ firing rate (F-C)", "△ SNB replay ratio (F-C)" respectively for clarity. F stands for the familiar pattern. C stands for control patterns.

4

Q5. Regarding statements about human memory capacity, the finding of Standing are probably a bit misleading. For more modern discussions of this issue see Vogt and Magnussen (2007) and Dudai (1997).

We appreciate the references provided. We have re-written the sentences below to incorporate the papers mentioned by the reviewer(s).

In Page 1 Line 2, "Humans have an almost "limitless" capacity of recognition memory (Standing, 1973)" to "Humans have a larger than expected capacity of recognition memory (Dudai, 1997)".

In Page 3 Line 6-8, "Prior studies (Shepard, 1967; Standing, 1973) have demonstrated that our recognition memory for images is highly accurate and has no practical capacity limit" to "Prior studies (Nickerson 1965; Shepard, 1967; Standing, 1973) have demonstrated that subjects can recognize many thousands of pictures several days after they have seen the pictures only once, suggesting a vast recognition memory for images, with no capacity limit detected in practical experiments".

The reference by Vogt and Magnussen (2007) is cited on Page 1 Line 4.

Q6. The description of CCH should be clarified regarding what i and j represent.

In the CCH equation, i, j are the time-series representations (1-dimensional vectors) of the stimulus time points (i) and the electrode response time points (j). The entire probing session was binned into 1-ms windows. For a particular stimulating position, if a probing stimulus is imposed in a particular time bin, the time-series vector i will contain 1 in the corresponding time bin, otherwise it is 0. For a particular electrode, if it detects a spike in a particular time bin, the time-series vector j will contain 1 in the corresponding time bin, otherwise 0. CCH (cross-correlation histogram) calculates the discrete cross-correlation between the two time-series and find the time lag that returns the largest correlation (the peak of the CCH curve). We regard the largest correlation with a positive time lag as an indicator for a potential causal relationship between the stimulating position (input) and the electrode (output), hence a potential connection. (The explanation above has been added into the Methods section.)

It would be helpful in figure 3B1, the labels of the lines were C1 and C2.

The labels have been changed to C1 and C2 in Figure 3B1.

Q7. There are a number of linguistic corrections that could be made. E.g., Li 262. "coined" do the authors mean "quantified"?

"Coined" is replaced by "quantified" in Line 262 (Page 13 Line 14).

5

Li. 353. "from here on" instead of "in future".

In Line 353 & 354, "in future" is replaced by "from here on" (Page 17 Line 13 & 14).

Li. 431. "could not be subsequently reproduced".

"Could not be reproduced in subsequent repeats" is replaced by "could not be subsequently reproduced" in Line 431 (Page 21 Line 2).
